# Intestinal Behcet’s syndrome with an unusual complication bladder-intestines fistula and urinary tract infections—A case report

**DOI:** 10.3389/fcimb.2023.1108247

**Published:** 2023-03-29

**Authors:** Jiewen Deng, Xuanming Fan, Hui Guo, Jie Gao, Xuan Li, Wei Wan

**Affiliations:** ^1^ The Department of Cardiovascular Diseases, The First Affiliated Hospital of the Naval Military Medical University, Shanghai, China; ^2^ The Department of Gastroenterology, The First Affiliated Hospital of the Naval Military Medical University, Shanghai, China; ^3^ Department of Pharmacy, Shanghai Tenth People’s Hospital, Tongji University School of Medicine, Shanghai, China; ^4^ Department of Rheumatology and Immunology, The First Affiliated Hospital of the Naval Military Medical University, Shanghai, China; ^5^ Shanghai Songjiang Fangta Hospital of TCM, Shanghai, China

**Keywords:** intestinal Behcet’s syndrome, perforation of the intestine, fistula formation and urinary tract infections, capsule endoscope, biological agents

## Abstract

A 33-year-old male patient with a 17-year Behcet’s syndrome history showed abdominal pain and fever symptoms. The abdominal CT was suggestive of an acute ileocecal intestinal perforation. In addition, the symptoms disappeared after the conservative treatment. Some related examinations, including capsule endoscopy, were performed to explain the phenomenon of the food residue urine. These results indicated the intestine-urinary tract fistula formation, supposed to be the outcome of intestinal Behcet’s syndrome perforation. This is a rare case of intestinal Behcet’s syndrome with abdominal symptoms as the main manifestation. It was complicated by entero-urinary fistula formation and urinary tract infections. Now, we report this story to emphasize that capsule endoscopy is conducive to the diagnosis and assessment of the intestinal Behcet’s syndrome; moreover, anti-inflammatory treatment including biological agents is effective to relieve the disease at the acute stage in addition to surgical methods.

## Introduction

Behcet’s syndrome is an idiopathic, chronic, relapsing, and multi-systemic vasculitis with the characteristic of recurrent oral and genital aphthous ulcers, ocular disease, and skin lesions. It was first described by the Turkish dermatologist Halushi Behçet in 1937 (Bulur and Onder, 2017; Hatemi et al, 2018). The highest prevalence of Behcet’s syndrome occurred in countries along the ancient Silk Road from the Mediterranean basin to East Asia, in contrast to the low prevalence in North American and Northern European countries. In addition, in China, the incidence of Behcet’s syndrome is about 14 per 100,000, and it more commonly and severely happened in young and middle-aged men than in women (Davatchi et al., 2017).

Gastrointestinal involvement rarely exists in Behcet’s syndrome, always easily overlooked. Gastrointestinal lesions induced by Behcet’s syndrome as a complication did not attract people’s attention until Jensen first reported the gastrointestinal manifestations of Behcet’s syndrome patients (Jensen and Donde, 1978). Gastrointestinal manifestations of Behcet’s syndrome are very important because of the association with significant morbidity and mortality (Skef et al., 2015). Also, sometimes, Behcet’s syndrome patients companied by gastrointestinal lesions are at high risk of malignant tumors (Jung et al., 2017). Diagnosis is mainly based on clinical criteria, because there are no pathognomonic laboratory tests. The methods for monitoring the disease activity, including digestive endoscopy, are available but imperfect. Our report described a case of an unusual complication bladder-intestines fistula with colonic perforation history resulting from intestinal involvement with the Behcet’s syndrome.

## Case report

**Figure 1:**
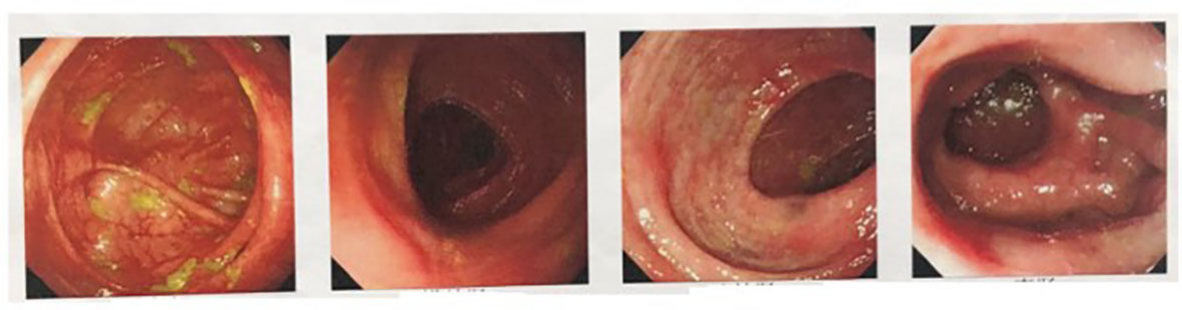
Multiple flaky erosion of the colon sigmoid colon, mucosal hyperemia, and edema.

**Figure 2:**
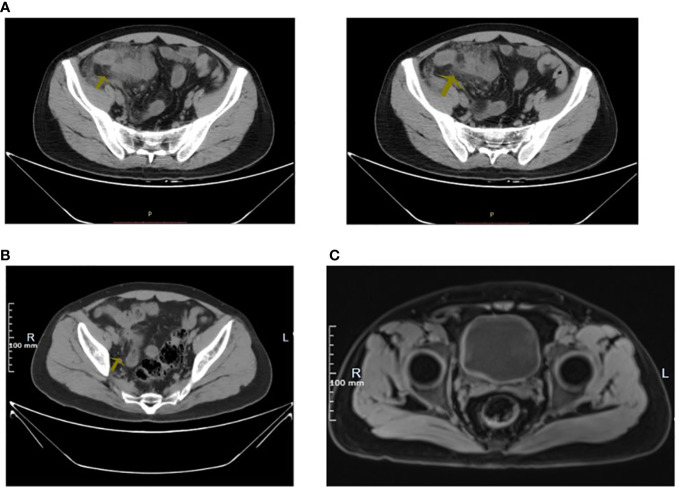
**(A)** Hypogastrium CT in July 2021: The arrow displayed that there was unclear appendix, the cloudy surrounding fat space, and a little free gas. **(B)** Hypogastrium CT in November 2022: The arrow displayed that there was slightly entangled intestine with the blurred surrounding fat space in the right pelvic cavity. **(C)** MRI: The arrow displayed that the bladder filled well.

A 33-year-old young male patient was admitted to the hospital owing to intermittent abdominal pain for 16 months and aggravated pain for 3 months. The patient has a 17-year history of recurrent oral and peptic ulcer pain. He was diagnosed with Behcet’s syndrome 17 years ago by the Department of Rheumatology and Immunology(The First Affiliated Hospital of Naval Medical University) because of “Painless oral ulcer and the gradual appearance of erythema nodosum on the face”. The rice grain-size red macules and papules appeared after aseptic acupuncture and subsided for 1 week. Meanwhile, no pustules were formed. Prednisone acetate tablets were given orally at 20 mg/day at first. After 3-month continuous administration, its dosage decreased to 10 mg/day. Then, the patient stopped the drug administration by himself after 6-month continuous treatment, because the symptoms improved. However, oral ulcers and esophageal ulcers recurrently and intermittently attack him during the 17 years. In 2010, he was admitted to the emergency department of Changhai Hospital because of “Hematochezia and general weakness”. He had taken 3-month prednisone acetate again at 20 mg/day after fluid replacement therapy owing to the rectal ulcer bleeding caused by Behcet’s syndrome. He stopped again taking medication when the symptoms improved. In May 2021, intermittent symptoms with fever, diarrhea, and mucinous stools without pus and blood entangled him. And then, the defecation frequency increased to three times every day. A colonoscopy was performed. The results showed multiple patchy erosion of the sigmoid colon and rectum (**Figure 1**). In July 2021, the patient suddenly presented with abdominal tenderness, rebound pain, and muscle tension after meal. He was referred to the emergency department and performed a CT scan of the lower abdomen. The patient was considered to have a perforation of the intestine with the hint of the progression of ileocecal ulcer according to his 17-year Behcet’s syndrome history (**Figure 2A**). The operative treatment for the intestinal segment resection was advised, but the patient refused it and accepted conservative anti-infection therapy (cefoperazone-sulbactam sodium, 2.0 g/day). Then, he got symptomatic relief and was discharged from the hospital post-treatment. In the past 3 months, from August 2022, the patient suffered from aggravated diffuse abdominal pain with intermittent low fever for 7 days and debris urine, while there was no nausea, vomiting, chills, high fever, and so forth. The abdominal pain eased off after rest.

**Figure 3:**
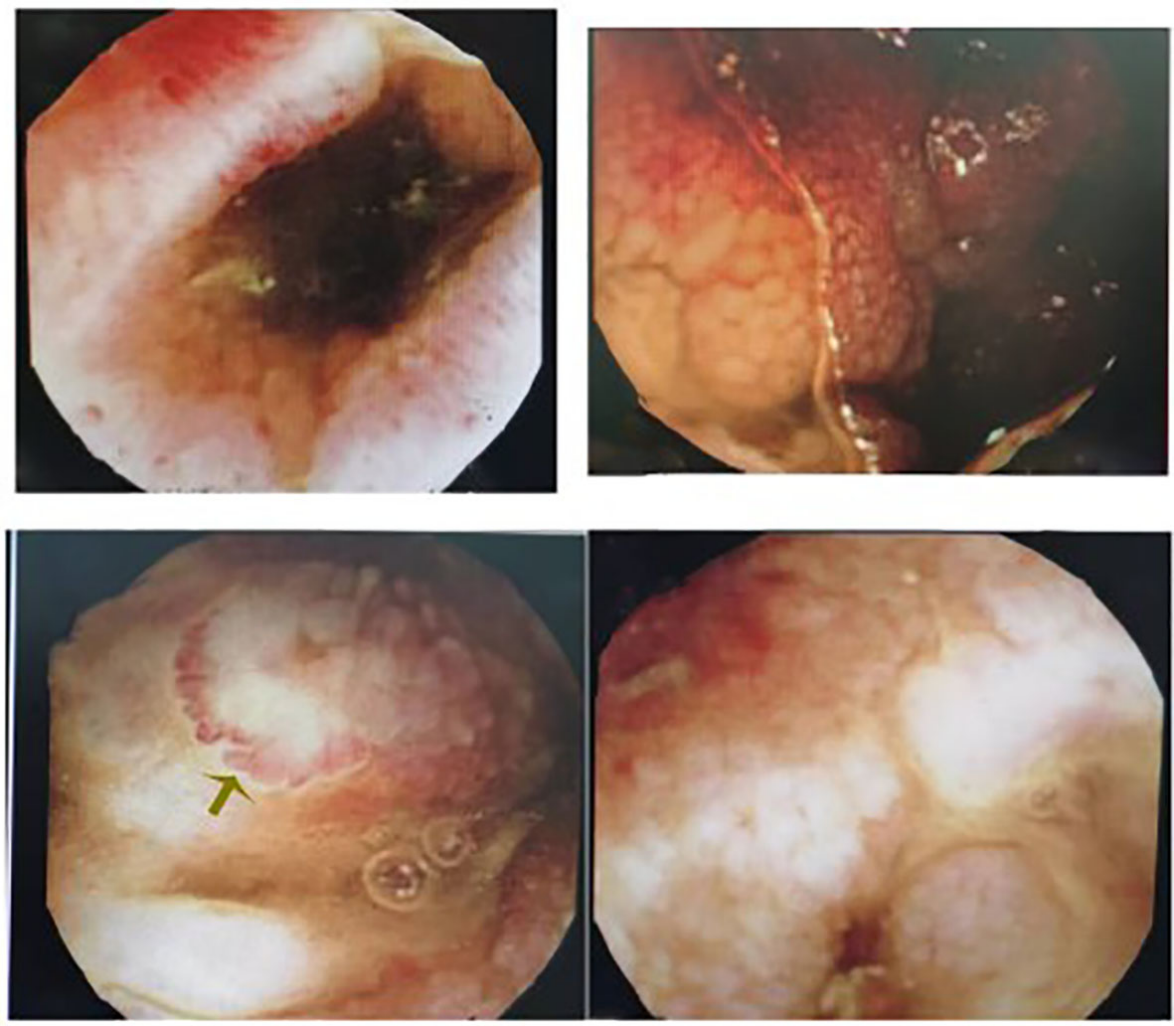
Capsule endoscope: The small intestinal mucosa has multiple flaky erosions and pitting lesions, with hyperemia and edema surrounding them.

To solve the problem of the debris urine, he asked for help from the rheumatism immunology department in November 2022, at the First Affiliated Hospital of Naval Medical University this time. The blood test report Leukocyte 5.3 × 10 (Cheon et al., 2009)/liter, hemoglobin 136 g/liter, ESR 2 mm/H, C-reactive protein 9.29 mg/liter, RNP(±), and other autoantibodies were negative. A plain CT scan of the lower abdomen showed a slight accumulation of the right small intestine in the pelvis, a vague surrounding adipose space, and suggested inflammation (**Figure 2B**). Bladder MRI prompted a well-filled bladder (**Figure 2C**). According to the urine routine examination, WBC 500.0/µl, and positive midstream urine culture (Klebsiella metazoans, a kind of Gram-negative bacteria settled in the intestine and respiratory tract), fistula formation was considered. Given mental and physical conditions, the painless capsule endoscopy was performed. The result of the capsule endoscopy demonstrated that there were multiple round erosive lesions and depressed lesions in the small intestinal mucosa, circular depressed lesions in the ileum mucosa, congestion, and edema in the peripheral mucosa (**Figure 3**). Meanwhile, no obvious active bleeding was observed (**Figure 3**). In terms of treatment, the patient received levofloxacin intravenous infusion to prevent infection, and adalimumab (40 mg, 1/2 week) subcutaneous injection for the cure of the primary disease—Behcet’s syndrome. After a 2-week treatment, the patient who got symptomatic relief was discharged from the hospital. Because of the rejection to get medical advice from Urology, the patient was advised to rheumatology outpatient follow-up.

## Discussion

According to statistics, only 3.3% proportion of Behcet’s syndrome patients appear with gastrointestinal discomfort as the first symptom and 8.8% proportion of Chinese Behcet’s syndrome patients get gastrointestinal involvement (Hatemi et al., 2016). In our case, the patient appeared with gastrointestinal symptoms and related extremely rare complications—”a small amount of undigested intestinal contents were found in the urine during fever”. It was considered as the formed corresponding fistula between the intestine and urinary tract, confirmed by an anorectal surgeon. The surgeon claimed that the symptom of debris urine provided robust evidence to certify the existence of a bladder-intestines fistula, and a cystoscope was recommended for treatment. However, the patient declined the advice.

Currently, the main examination method for early diagnosis of intestinal Behcet’s syndrome is digestive endoscopy. A meta-analysis showed that capsule endoscopy was more suitable for bleeding lesion detection than conventional methods, with 87% detection rate of intestinal Behcet’s syndrome. In our case, capsule endoscopy with the advantage of painlessness and safety is recommended for Behcet’s syndrome patients with gastrointestinal manifestations, whether mild or not, to assess the severity of the lesion. The 2022 consensus on the diagnosis and treatment of intestinal Behcet’s syndrome points out that the most typical presentation is a single, large, and well-circumscribed ulcer in the ileocecal region (Cheon et al., 2009; Lee et al., 2009; Chung et al., 2010). In our case, the isolated, oval, and single small intestinal ulcers under capsule endoscopy (Cheon et al., 2009; Lee et al., 2009; Chung et al., 2010) (**Figure 3**) and the more-than-10-year diagnosis history of Behcet’s syndrome supported the intestinal Behcet’s syndrome diagnosis.

The incidence of ileocecal region ulcers ranked first, followed by the appearance of esophageal ulcers. As the intestinal Behcet’s syndrome ulcers process, complications such as perforation, intestinal obstruction, and massive gastrointestinal bleeding may occur (Turan et al., 2003; Dowling et al., 2008), and some kinds of ulcers may show signs of healing with the development of the disease (Chung et al., 2010). The patient had a bowel perforation history, supposed to receive surgical treatment theoretically. Miraculously, after conservative treatment (glucocorticoids and antibiotics), he was discharged from the hospital without resection of the intestine segment. In other words, surgical treatment of intestinal Behcet’s syndrome is not advocated sometimes because of the repetitive inflammation, considering the poor prognosis. Although the indications of emergency surgery for intestinal Behcet’s syndrome include intestinal perforation, massive bleeding, intussusception, and intestinal obstruction (Hatemi et al., 2016), the treatment for intestinal Behcet’s syndrome perforation requires multidisciplinary consultation and deserves to be studied further. In our case, the comparison of the two different CT results (**Figure 2A** CT in July 2021 and **Figure 2B** CT in November 2022) suggested that the intestinal perforation area gradually healed, which was extremely rare. In addition, although the MRI showed a well-filled bladder (**Figure 2C**), the patient had debris urine when he had a fever, with some positive examinations including the elevated urine leukocytes and positive midstream urine cultures (Gram-negative bacteria always settled in the intestine). It may result from the gradual tissue healing and regeneration of the fistula after corresponding anti-inflammatory treatment, or the pathological changes into chronic proliferative inflammation, also remaining to be further explored. Moreover, the fistula needs repair by cystoscopy when intestinal Behcet’s syndrome was controlled and stable for 3 months. Glucocorticoid and immunosuppressive therapies are necessary for postoperative wound healing and prevention of recurrence (Hatemi et al., 2016).

Corticosteroids, 5-aminosalicylic acid derivatives, immunomodulators, blood transfusions, thalidomide, pentoxifylline and, more recently, antitumor necrosis factor monoclonal antibody have all been used to treat Behcet’s syndrome with varying degrees of success (Kasahara et al., 1981; Hassard et al., 2001; Bettiol et al., 2020). According to the 2020 Chinese expert consensus on the diagnosis and treatment of Behcet’s syndrome, anti-TNF monoclonal antibodies and/or thalidomide can be used for severe and/or refractory patients. In our case, the patient was treated with Adalimumab, the promising drug for the medicine of refractory Behcet’s syndrome with its determining efficacy (Tanida et al., 2015; Ueda et al., 2015).

## Data availability statement

The original contributions presented in the study are included in the article/supplementary material. Further inquiries can be directed to the corresponding author.

## Ethics statement

Written informed consent was obtained from the individual(s) for the publication of any potentially identifiable images or data included in this article.

## Author contributions

JD and XF examined and treated the patient. WW designed and supervised this study. JD, XF, HG and JG wrote the manuscript. XL checked the manuscript. All authors contributed to the article and approved the submitted version.
